# SGLT2 inhibitor dapagliflozin prevents atherosclerotic and cardiac complications in experimental type 1 diabetes

**DOI:** 10.1371/journal.pone.0263285

**Published:** 2022-02-17

**Authors:** Judit Hodrea, Adar Saeed, Agnes Molnar, Attila Fintha, Adrienn Barczi, Laszlo J. Wagner, Attila J. Szabo, Andrea Fekete, Dora B. Balogh

**Affiliations:** 1 MTA-SE “Lendület” Diabetes Research Group, Semmelweis University, Budapest, Hungary; 2 1^st^ Department of Pediatrics, Semmelweis University, Budapest, Hungary; 3 1^st^ Department of Pathology and Experimental Cancer Research, Semmelweis University, Budapest, Hungary; 4 Department of Transplantation and Surgery, Semmelweis University, Budapest, Hungary; 5 ELKH-SE Pediatrics and Nephrology Research Group, Hungarian Academy of Sciences and Semmelweis University, Budapest, Hungary; Temple University, UNITED STATES

## Abstract

**Introduction:**

Cardiovascular disease (CVD) is two to five times more prevalent in diabetic patients and is the leading cause of death. Therefore, identification of novel therapeutic strategies that reduce the risk of CVD is a research priority. Clinical trials showed that reduction in the relative risk of heart failure by sodium-glucose cotransporter 2 inhibitors (SGLT2i) are partly beyond their glucose lowering effects, however, the molecular mechanisms are still elusive. Here we investigated the role of SGLT2i dapagliflozin (DAPA) in the prevention of diabetes-induced cardiovascular complications.

**Methods:**

Type 1 diabetes was induced with streptozotocin (65 mg/bwkg, *ip*.) in adult, male Wistar rats. Following the onset of diabetes rats were treated for six weeks with DAPA (1 mg/bwkg/day, *po*.).

**Results:**

DAPA decreased blood glucose levels (D: 37±2.7 *vs*. D+DAPA: 18±5.6 mmol/L; p<0.05) and prevented metabolic decline. Aortic intima-media thickening was mitigated by DAPA. DAPA abolished cardiac hypertrophy, and myocardial damage. Cardiac inflammation and fibrosis were also moderated after DAPA treatment.

**Conclusions:**

These data support the preventive and protective role of SGLT2i in diabetes-associated cardiovascular disease. SGLT2i may provide novel therapeutic strategy to hinder the development of cardiovascular diseases in type 1 diabetes, thereby improve the outcomes.

## Introduction

Cardiovascular disease (CVD) and related mortality are increasing at an alarming rate, also as a consequence of growing prevalence of diabetes mellitus (DM) [1]. About two-thirds of deaths in people with diabetes are due to CVD including mainly ischemic heart disease, but also congestive heart failure (HF) and stroke [2]. The impact of type 1 DM (T1DM) and type 2 DM (T2DM) on CVD mortality is similar, however T1DM patients are younger at the onset of the disease, thus they lose more life-years due to CVD [3, 4]. Impaired glucose metabolism, inflammation, and cell signaling abnormalities lead to premature atherosclerosis, myocardial remodeling, and left ventricular (LV) fibrosis contributing to cardiovascular damage. So far, commonly applied glucose-lowering agents have had little or no impact on CVD progression and outcomes in diabetic patients. Therefore, identifying novel therapeutic strategies targeting cardiovascular inflammation and fibrosis is of paramount importance in the management of CVD especially in T1DM.

Sodium-glucose cotransporter 2 inhibitors (SGLT2i) represent a novel class of antidiabetic drugs mainly used in T2DM. To date, dapagliflozin (DAPA) is the only approved selective SGLT2i in adults with T1DM in the European Union. Considerable advantage of SGLT2i is that they work independently of insulin by blocking proximal tubular glucose reabsorption resulting in reduced plasma glucose level and glucosuria. Large-scale clinical trials: EMPA-REG OUTCOME, CANVAS and DECLARE-TIMI 58 [5–7] have been evaluated the selective SGLT2i empagliflozin, canagliflozin, and DAPA for their cardiovascular safety. Each of these trials were associated with reduction in the relative risk of HF indicating a class effect of SGLT2i. Moreover, the DAPA-HF study reported that DAPA was more efficient compared to placebo in the prevention of cardiovascular death and HF, regardless of the presence or absence of DM [8]. Further, based on the DAPA-HF trial the US Food and Drug Administration (FDA) has recently approved DAPA to reduce the risk of cardiovascular death in adults with heart failure with reduced ejection fraction [9]. These data suggest that the cardioprotective effects of SGLT2i cannot be solely attributed to the glucose lowering action.

Elevated levels of inflammatory mediators are associated with hyperglycemia, insulin resistance, and may predict the development of DM [10]. Growing body of evidence revealed that chronic inflammation is a major driver of diabetic cardiomyopathy (DCM) [11, 12]. Accordingly, inflammation could be a possible common mechanism in the pathophysiology of both DM and CVD. Glucotoxicity and dyslipidemia directly induce upregulation and secretion of inflammatory mediators and contribute to atherosclerosis, cardiac hypertrophy, remodeling, fibrosis and may interfere with cardiomyocyte contractile properties [13]. Recent studies demonstrated that empagliflozin and DAPA reduce cardiac interstitial macrophage infiltration in prediabetic rats and in infarcted rat hearts, decrease mRNA expressions of interleukin-1β (IL-1β), interleukin-6 (IL-6) and mitigate the activation of Nlrp3/ASC inflammasome, thus attenuate the development of DCM in T2DM mouse model [14–16]. However, literary data is scanty about the anti-inflammatory role of SGLT2i in T1DM.

Bearing in mind the high relative risk of death from CVD in T1DM and the enormous importance of SGLT2i as possible cardioprotective agents in T2DM, it is important to investigate whether DAPA can mitigate the development of DCM in T1DM. In the present study, DAPA was applied to test its anti-inflammatory properties in the prevention of atherosclerosis and cardiac fibrosis in a streptozotocin-induced T1DM rat model.

## Materials and methods

### Study approval

All experiments were conducted in accordance with guidelines of the Committee on the Care and Use of Laboratory Animals of the Semmelweis University Budapest, Hungary (PEI/001/1731-9-2015).

### Materials

All chemicals and reagents were purchased from Sigma-Aldrich (St. Louis, MO, USA), and all standard plastic laboratory equipment was purchased from Sarstedt (Numbrecht, Germany) unless otherwise stated.

### Animals and study design

Experiments were performed on eight-week-old male Wistar rats (*Rattus norvegicus*) purchased from Toxi-Coop (Budapest, Hungary). Rats were placed in groups of three per cage under controlled light and temperature (12:12 hour light-dark cycle at 24±2°C) with *ad libitum* access to standard rodent diet and tap water. Rats underwent an acclimatization period for one-week before the commencement of the experiment. The health and well-being of the animals were monitored daily by a qualified person. Refined aspects of housing, husbandry, enrichment, and socialization were applied to prevent or alleviate distress (the number of the animals used for the experiment was reduced to the absolute minimum necessary, harming ambient light, noise, vibrations, fluctuations or extremes in temperature were absent; environment enrichment and more frequent, gentle and predictable handling were applied; husbandry handling was combined with habituation and handling for research purposes).

T1DM was chemically induced with a single intraperitoneal injection of 65 mg/bwkg streptozotocin (STZ) dissolved in 0.1 M citrate buffer (pH 4.5) after overnight fasting. Blood glucose levels were measured three times from tail vein with a D-Cont Ideal device (77 Elektronika, Budapest, Hungary). Rats with a peripheral blood glucose value above 15 mmol/L 72 hours after the STZ injection were enrolled in the study. Diabetic (D) rats were randomly divided into two groups immediately after the onset of diabetes (n = 6/groups) and were treated *per os* as follows: (i) isotonic saline (NaCl 154 mmol/L) as vehicle (D) and (ii) DAPA dissolved in isotonic saline (D+DAPA; 1 mg/bwkg/day). Age-matched non-diabetic controls (C) received the equivalent volume of citrate buffer without STZ once, and the same amount of saline by oral gavage daily at the same time as the diabetic animals during the six-week study period (n = 6 /group). To prove the safety of the treatment, an additional DAPA-treated non-diabetic control group (C+DAPA, 1 mg/bwkg/day) was investigated only for laboratory parameters. These results are presented as Supporting Information. Blood pressure, serum and urinary parameters were determined at the end of the study period. Rats were placed into metabolic cages to collect urine for a 24-hour period before euthanasia. At the end of the protocol rats were euthanised using a mixture of 75 mg/bwkg ketamine (Richter Gedeon, Budapest, Hungary) and 10 mg/bwkg xylazine (Medicus Partner, Biatorbagy, Hungary).Blood, urine, and tissue samples were collected, weighed, and stored for further investigations.

### Measurement of arterial blood pressure

A CODA tail-cuff standard monitoring system (EMKA Technologies, Paris, France) was used to measure the systolic and diastolic blood pressures, which is based on volume pressures recording and is a clinically validated technology. Mean arterial pressure (MAP) was calculated. The recording was performed in a suitable environment without any distractions. Rats were adapted for 10 min/day to the measurement in 2 days before the recording started. Subsequently, rats stayed quiet and remained in the holder on the testing day.

### Measurement of metabolic parameters

Serum and urine samples were measured with available standard kits on a Hitachi 912 photometric chemistry analyzer (Roche Hitachi, Basel, Switzerland). Glucosuria was determined from 24-hour collected urine.

### Measurement of klotho and cardiac troponin I

Serum klotho and cardiac troponin I were measured according to the manufacturer’s protocol using Rat Klotho ELISA Kit (ABclonal, Woburn, MA, USA) and Rat Cardiac Troponin I ELISA Kit (Abcam, Cambridge, UK). 96-well microplates were analyzed with SPECTROstar Nano microplate reader (BMG Labtech, Ortenberg, Germany) at 450 nm with 650 nm as reference.

### Histology

Amount of elastic fibers were investigated on orcein stained aorta sections. The aorta was dissected under a light microscope, immediately fixed in 8% paraformaldehyde, and embedded in paraffin for immunohistochemistry. 5 μm sections were deparaffinized in xylene and rehydrated (100%, 90%, 70% ethanol, and distilled water). Sections were immersed in 1% orcein at 60°C for 30 min, followed by differentiation in acid-alcohol (1:99 hydrochloric acid and 70% ethanol) for 10 seconds to remove the dye excess, and then into distilled water. Histological examination was performed under 20x objective magnification using Case Viewer 2.4. (3DHISTECH, Budapest, Hungary). Intima-media thickness (IMT) was measured on cross sections of the aorta and the mean value of ten measurements were calculated.

Collagen content and perivascular fibrosis of the left ventricle was determined with picrosirius red staining. Fresh frozen heart tissues were sectioned with 4 μm thickness, slides were stained, and were digitalized with a Pannoramic1000 slide scanner (3DHistech, Budapest, Hungary). The slides were visualized with SlideViewer2.5 (3DHistech, Budapest, Hungary), and intramyocardial collagen represented red-colored area was measured with the Quant Center HistoQuant 2.5 module (3DHistech, Budapest, Hungary). The myocardial field of measurement was manually selected with the caution of avoiding the endocardium and perivascular connective tissue. The surface of measurement was 1,6 mm^2^ in each heart. The red-stained area was measured based on the red color intensity. First, multiple optimalization measurements were performed with the supervision of an experienced cardiovascular histopathologist, reaching the best RGB pixel intensity parameters: Red channel 84–221, Green channel 8–142, Blue channel 10–137. Then the red-stained area measurements were performed on each histological slide.

### Quantitative RT-PCR

Total RNA was extracted using the Total RNA Mini Kit (Geneaid Biotech, New Taipei City, Taiwan). Measurement of quality and quantity of isolated RNA was performed by NanoDrop ND-1000 spectrophotometer (Baylor College of Medicine, Houston, TX, USA). RNA (500 ng) was reverse-transcribed using the Maxima^™^ First Strand cDNA Synthesis Kit for RT-qPCR (Thermo Fisher Scientific, Waltham, MA, USA) to generate first-strand cDNA. Atrial natriuretic peptide (*Nppa*), B-type natriuretic peptide (*Nppb*), transforming growth factor beta (*Tgfb1*), platelet-derived growth factor (*Pdgfb*), connective tissue growth factor (*Ccn2*), plasminogen activator inhibitor 1 (*Serpine1*), fibronectin (*Fn*), interleukin-1β (*Il1b*), interleukin-6 (*Il6*), tumor necrosis factor alpha (*Tnf*), monocyte chemoattractant protein 1 (*Ccl2*), and 18S ribosomal RNA (*Rn18S*) were determined by using LightCycler 480 SYBR Green I Master enzyme mix (Roche Diagnostics, Indianapolis, IN, USA) and specific primers listed in Table 1. Results were analyzed by LightCycler^®^ 480 software (version 1.5.0.39, Roche Diagnostics). Target gene expressions were normalized against *Rn18S* as a housekeeping gene.

**Table 1:** Sequences of primer pairs for quantitative RT-PCR.

Gene	NCBI reference	Primer pairs	Product length (bp)
*Nppa*	NM_012612.2	Forward: 5’ CCTGGACTGGGGAAGTCAAC 3’ Reverse: 5’ GCAGCTCCAGGAGGGTATTC 3’	326
*Nppb*	NM_031545.1	Forward: 5’ TGACGGGCTGAGGTTGTTTT 3’ Reverse: 5’ ACACTGTGGCAAGTTTGTGC 3’	198
*Il1b*	NM_031512.2	Forward: 5’ GACTTCACCATGGAACCCGT 3’ Reverse: 5’ GGAGACTGCCCATTCTCGAC 3’	104
*Il6*	NM_012589.2	Forward: 5’ AGCGATGATGCACTGTCAGA 3’ Reverse: 5’ TAGCACACTAGGTTTGCCGA 3’	409
*Tnf*	NM_012675.3	Forward: 5’ ACTGAACTTCGGGGTGATCG 3’ Reverse: 5’ GCTTGGTGGTTTGCTACGAC 3’	153
*Ccl2*	NM_031530.1	Forward: 5’ GATCCCAATGAGTCGGCTGG 3’ Reverse: 5’ ACAGAAGTGCTTGAGGTGGTT 3’	294
*Tgfb1*	NM_021578.2	Forward: 5’ GCACCGGAGAGCCCTGGATACC 3’ Reverse: 5’ CCCGGGTTGTGTTGGTTGTAGAGG 3’	222
*Pdgfb*	NM_031524.1	Forward: 5’ TCGATCGCACCAATGCCAACTTCC 3’ Reverse: 5’ CACGGGCCGAGGGGTCACTACTGT 3’	236
*Ccn2*	NM_022266.2	Forward: 5’ TCCACCCGGGTTACCAATGACAATAC 3’ Reverse: 5’ CTTAGCCCGGTAGGTCTTCACACTGG 3’	195
*Serpine1*	NM_012620.1	Forward: 5’ CCTCCTCATCCTGCCTAAGTT 3’ Reverse: 5’ CTTGACCTTTTGTAGTGCTTGTG3’	163
*Fn*	NM_019143.2	Forward: 5’ GGATCCCCTCCCAGAGAAGT 3’ Reverse: 5’ GGGTGTGGAAGGGTAACCAG 3’	188
*Rn18S*	NR_046237.1	Forward: 5’ GCGGTCGGCGTCCCCCAACTTCTT 3’ Reverse: 5’ GCGCGTGCAGCCCCGGACATCTA 3’	105

### Western blot analysis

All chemicals and reagents for Western blot analysis were purchased from Bio-Rad Laboratories (Hercules, CA, USA). Total protein was extracted from the left ventricle of the heart. Samples were homogenized in lysis buffer by TissueLyser LT homogenisator (Qiagen, Hilden, Germany). Protein concentrations were measured and mixed with 4x Laemmli sample buffer (1:3) and heated at 95°C for 5 minutes. Appropriate amount of proteins were electrophoretically resolved on polyacrylamide gradient gels (4–20%), then transferred to nitrocellulose membranes. The protein transfer was verified by 1% Ponceau S staining. After blocking, membranes were immunoblotted with specific primary antibody: α-SMA (A2547; Sigma Aldrich; 1:500). After repeated washing, the blots were incubated with the appropriate horseradish peroxidase (HRP)-conjugated secondary antibodies. Luminata^TM^ Forte Western HRP Substrate (Millipore Corporation, Billerica, MA, USA) was used for chemiluminescent detection of blots. Densitometric analysis of bands was performed by Quantity One Analysis software (Bio-Rad Laboratories). After background subtraction, integrated optical densities of bands of interest were factored for Ponceau S staining to correct for variations in total protein loading. Each blot was normalized to an internal control so that bands on separate blots could be compared.

### Statistical analysis

Data are expressed as means±standard deviations (SD). Statistical analysis was performed using Prism software (version 7.0; GraphPad Software, San Diego, CA, USA). Multiple comparisons and interactions were evaluated by one-way ANOVA followed by Holm-Sidak post hoc test. For non-parametrical data, the Kruskal-Wallis ANOVA on ranks followed by with Dunn’s correction was used. P values of <0.05 were considered significant.

## Results

### DAPA prevents metabolic decline in diabetic rats

Typical metabolic features of DM such as weight loss, elevated serum glucose, fructosamine, and lipid levels developed after 6 weeks of T1DM. As expected, DAPA markedly improved all metabolic parameters, while had no effect in non-diabetic control rats except inducing glucosuria (S1 Table). Dysregulation of carbohydrate metabolism was ameliorated in DAPA-treated diabetic rats, which was confirmed by the decrement of serum glucose and fructosamine levels. In parallel, DAPA enhanced the urinary glucose excretion in both control and diabetic groups confirming the mechanism of action of SGLT2i. Diabetes-induced dyslipidemia was normalized to control levels in DAPA-treated rats (Table 2).

**Table 2:** Dapagliflozin treatment improves T1DM-induced metabolic changes.

Metabolic parameters	Control (C)	Diabetic (D)	D+DAPA
Body weight (g)	442±35.4	256±29.9***	340±35.0^§§^
Non-fasting glucose (mmol/L)	6.42±0.58	33.3±1.06***	16.2±5.29***^§§§^
Fructosamine (μmol/L)	143±3.74	277±12.3***	198±34.9^§§§^
Total cholesterol (mmol/L)	1.96±0.15	2.82±0.30***	1.89±0.40^§§§^
Triglycerides (mmol/L)	1.24±0.51	3.12±1.17**	1.00±0.51^§§§^
LDL-C (mmol/L)	0.44±0.15	0.84±0.11***	0.48±0.13^§§§^
GOT (U/L)	127±19.6	382±164***	187±24.1^§§^
GPT (U/L)	43.0±8.39	181±82.1***	80.1±16.1^§§^
Glucosuria	UN	346±47.1***	491±94.2***^§§^

C, Control; D, diabetic; D+DAPA, dapagliflozin-treated diabetic; LDL-C, low-density lipoprotein cholesterol; GOT, serum glutamate-oxaloacetate transaminase; GPT, serum glutamate-pyruvate transaminase; *p* values indicate means±SDs and data were analyzed by one-way ANOVA with Holm-Sidak multiple comparisons test (n = 6 /group).

**p<0.01 *vs*. Control

***p<0.001 *vs*. Control

§§p<0.01 *vs*. Diabetic

§§§p<0.001 *vs*. Diabetic UN: undetectable.

### Cardiac hypertrophy and reduction in heart rate are hindered by DAPA

Systolic and diastolic pressure and heart rate were measured, mean arterial pressure (MAP) was calculated. After six weeks of DM, MAP remained unaltered in all groups. In line with the literature and other previous studies, STZ-induced DM was associated with reduction in heart rate [17, 18], which was reversed by DAPA treatment.

Cardiac hypertrophy developed in diabetic rats as indicated by increased heart to body weight ratio. DAPA treatment prevented cardiac hypertrophy (Table 3). Since all investigated parameter were the same in DAPA-treated non-diabetic controls (S1 and S2 Tables), further molecular research was performed only in control, diabetic and DAPA-treated diabetic rats.

**Table 3:** Mean arterial pressure, heart rate and heart to body weight ratio.

	Control	Diabetic (D)	D+DAPA
Mean arterial pressure (mmHg)	88.5±3.66	85.9±4.99	77.1±5.69
Heart rate (bpm)	444±12.9	320±25.2***	352±28.3^§^
Heart to body weight ratio (%)	0.29±0.01	0.36±0.02***	0.33±0.02^§§^

C, Control; D, diabetic; D+DAPA, dapagliflozin-treated diabetic.

Values indicate means±SDs and data were analyzed by one-way ANOVA with Holm-Sidak multiple comparisons test (n = 6/group).

***p<0.001 *vs*. Control

§p<0.05 vs. Diabetic

§§p<0.01 vs. Diabetic.

### Intima-media thickening is prevented by DAPA

**Fig 1:**
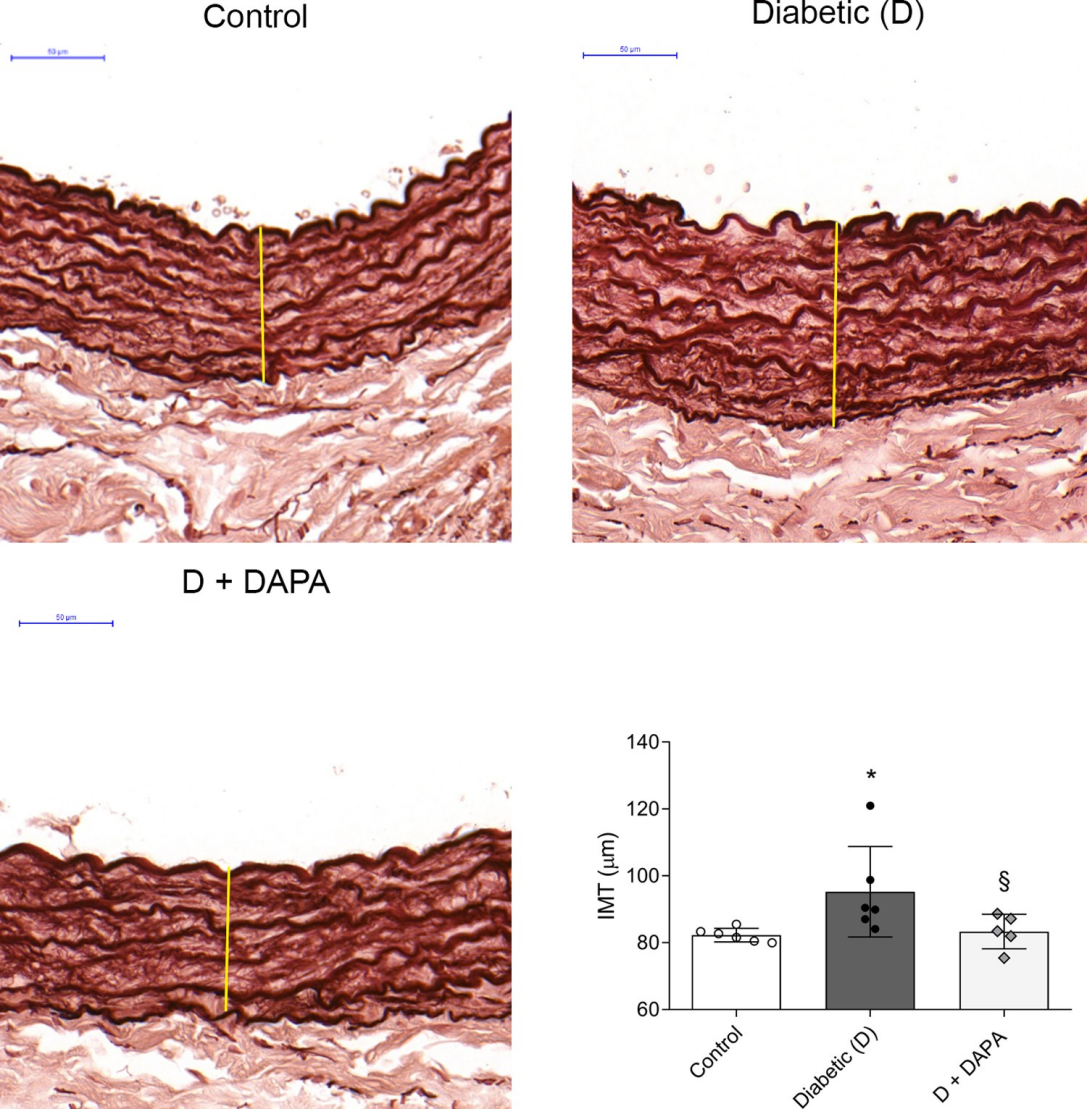
Intima-media thickening is prevented by dapagliflozin. Representative orcein stained aorta sections and quantitative evaluation of intima-media thickness (IMT) of control, diabetic (D) and dapagliflozin treated diabetic rats (D+DAPA). Original magnification, x200. Scale bar, 50 μm. Elastic fibers are stained brown after orcein staining. They are visualized as either thin fibers or elastic lamella. Bars indicate means±SDs and data were analyzed by one-way ANOVA with Holm-Sidak multiple comparisons test (n = 6/group). *p<0.05 *vs*. Control, ^§^p<0.05 *vs*. Diabetic.

Aortic IMT is an early marker of atherosclerosis. Changes in aortic IMT occur at early stages of the progression of T1DM and these become more severe with the duration of the disease [19, 20]. Histological examination of aortic IMT showed prominent wavy internal elastic lamina in control rats. Aorta of diabetic rats showed intimal thickening, irregularity and diffused elastic membranes, which was prevented by DAPA (Fig 1).

### DAPA mitigates the elevation of specific biomarkers of myocardial injury

**Fig 2:**
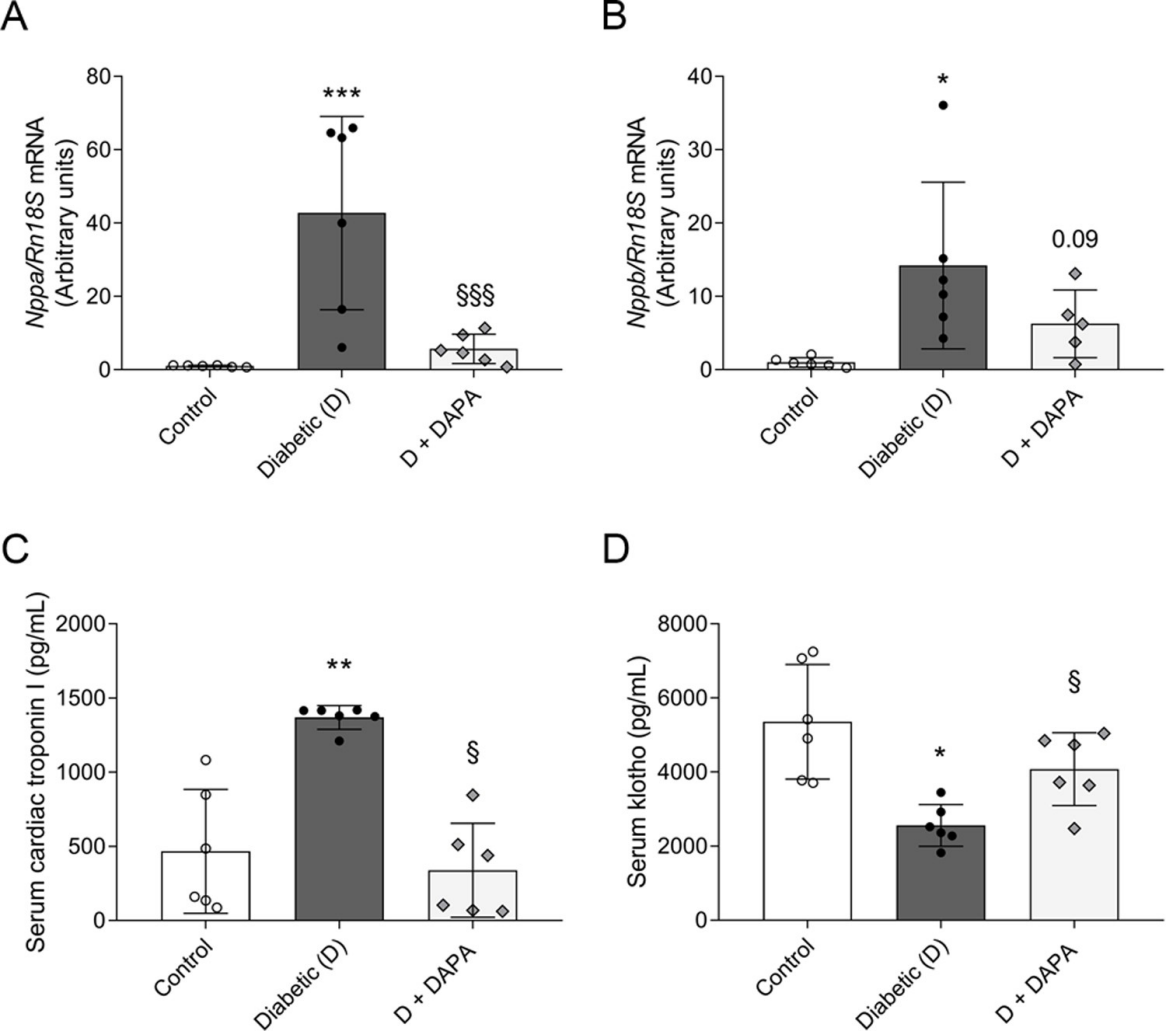
Diabetes-induced myocardial injury biomarker increment is halted by dapagliflozin. mRNA expression of (A) atrial natriuretic peptide (*Nppa*) and (B) B-type natriuretic peptide (*Nppb*) of control, diabetic and dapagliflozin treated diabetic rats (D+DAPA). mRNA expressions were normalized to *Rn18S* mRNA expression. (C) Serum levels of cardiac troponin I. (D) Serum levels of klotho. Bars indicate means±SDs and data were analyzed by one-way ANOVA with Holm-Sidak multiple comparisons test or Kruskal-Wallis with Dunn comparison test (n = 6/group). *p<0.05 *vs*. Control, **p<0.01 *vs*. Control, ***p<0.001 *vs*. Control, ^§^p<0.05 *vs*. Diabetic, ^§§§^p<0.001 *vs*. Diabetic.

Atrial natriuretic peptid (ANP) is considered as a marker for myocardial hypertrophy. It is mainly produced in the atria, but it is present in the ventricles as well. B-type natriuretic peptid (BNP) has been proposed as a useful biomarker for the determination of acute and chronic LV dysfunction, thus HF. BNP is predominantly released in response to LV volume expansion and pressure overload [21]. Therefore, mRNA expressions of *Nppa* (ANP) and *Nppb* (BNP) were measured in the LV. *Nppa* and *Nppb* were robustly elevated in the left ventricle of diabetic rats. Levels of both *Nppa* and *Nppb* were less elevated in DAPA-treated group (Fig 2A and 2B).

Cardiac troponins (cTn) received international approval as myocardial injury biomarker [22]. cTnI regulatory protein controls calcium-mediated interaction between actin and myosin and is a clinical indicator of myocardial cell damage [23]. Here we showed that DM-induced serum level of cTnI was decreased in DAPA-treated group (Fig 2C).

Klotho is a putative anti-aging protein, and it forms a unique endocrine system that regulates multiple metabolic processes (e.g., oxidative stress, inflammation, fibrosis). Here we found that klotho was downregulated in the serum of T1DM rats, which was halted by DAPA treatment (Fig 2D).

### Diabetes-induced cardiac inflammation is diminished by DAPA treatment

**Fig 3:**
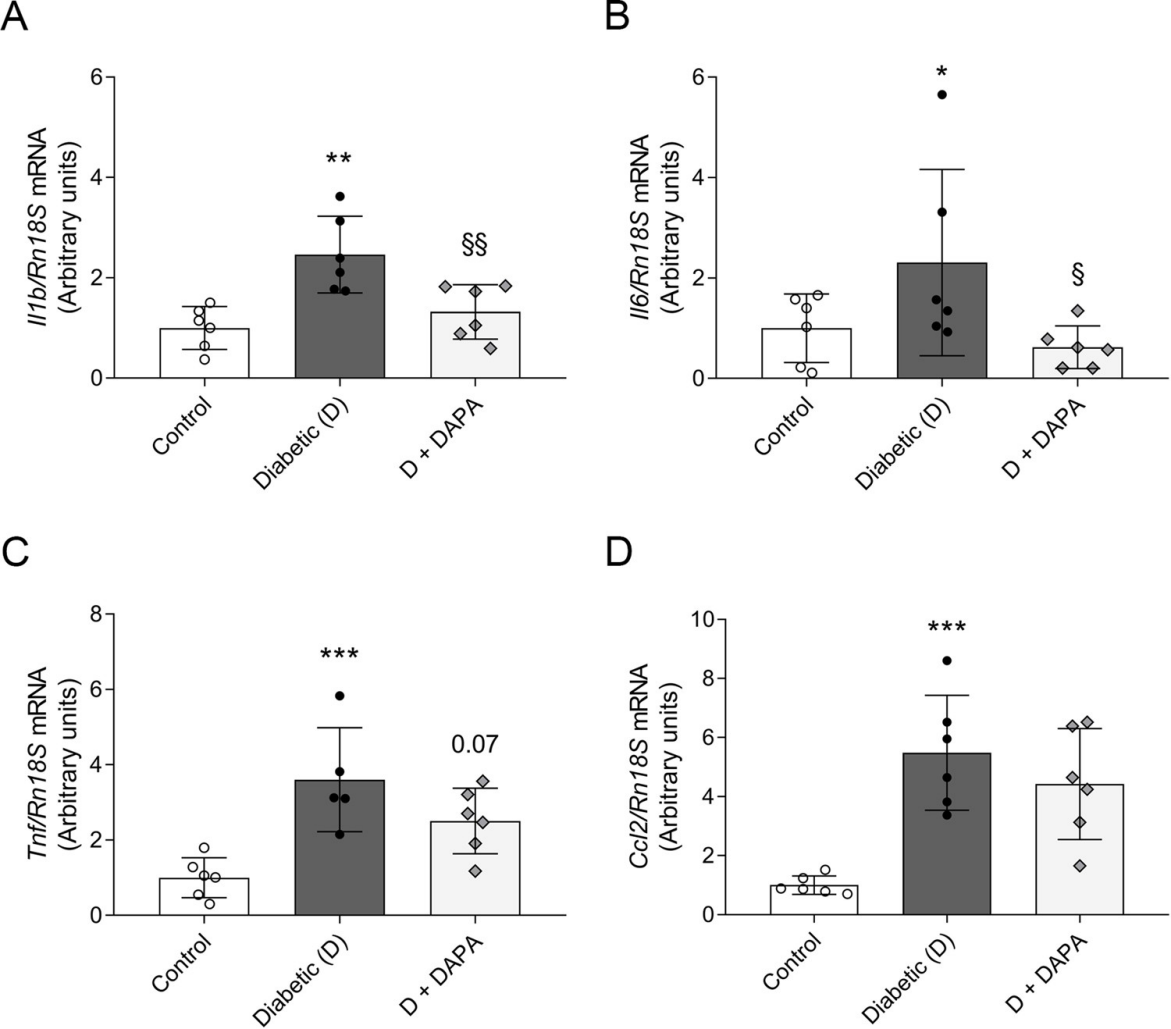
Dapagliflozin mitigates pro-inflammatory response in diabetic heart rats. mRNA expression of (A) interleukin-1β (*Il1b*), (B) interleukin-6 (*Il6*), (C) tumor necrosis factor (*Tnf*), and (D) monocyte chemoattractant protein 1 (*Ccl2*) of control, diabetic and dapagliflozin treated diabetic rats (D+DAPA). mRNA expressions were normalized to *Rn18S* mRNA expression. Bars indicate means±SDs and data were analyzed by one-way ANOVA with Holm-Sidak multiple comparisons test (n = 6/group) *p<0.05 *vs*. Control, **p<0.01 *vs*. Control, ***p<0.001 *vs*. Control, ^§^p<0.05 *vs*. Diabetic, ^§§^p<0.01 *vs*. Diabetic.

Chronic inflammation has been implicated in the pathogenesis of DCM. Cardiac overexpression of TNF-α, and IL-6 has been associated with myocardial damage, hypertrophy and fibrosis, as well with LV dysfunction. Accordingly, to investigate the anti-inflammatory properties of DAPA, *Il1b*, *Il6*, *Tnf* and *Ccl2* (MCP-1) mRNA expressions were measured in the left ventricle. All investigated cytokines and chemokine expressions were elevated in diabetic rats. Here we showed that DM-induced *Il1b*, *Il6 and Tnf* increment were abolished in DAPA treated group suggesting less severe inflammation induced myocardial damage (Fig 3A–3D).

### Progression of myocardial fibrosis is suspended in DAPA-treated diabetic rats

**Fig 4:**
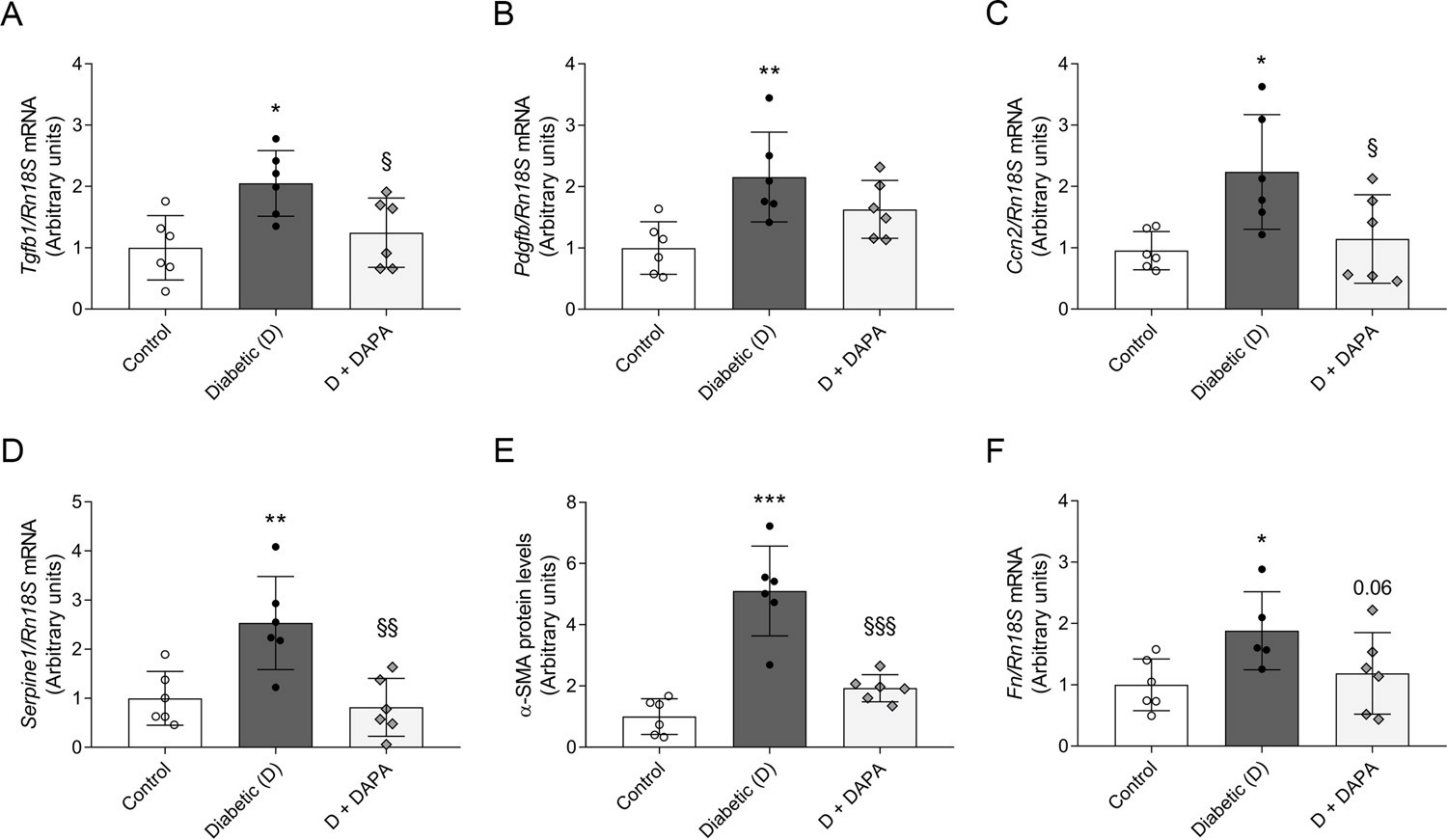
Dapagliflozin attenuates fibrosis in the left ventriculum of diabetic rats. mRNA expression of (A) transforming growth factor β (*Tgfb1*), (B) platelet-derived growth factor (*Pdgfb*), and (C) connective tissue growth factor (*Ccn2*) of control, diabetic and dapagliflozin treated diabetic rats (D+DAPA). (D) mRNA expression of *Serpine1* (also known as plasminogen activator inhibitor-1). (E) Protein level of α-smooth muscle actin (α-SMA). Proteins were normalized to total protein Ponceau S staining as loading control. (F) mRNA expression of fibronectin *(Fn)*. mRNA expressions were normalized to *Rn18S* mRNA expression. Bars indicate means±SDs and data were analyzed by one-way ANOVA with Holm-Sidak multiple comparisons test (n = 6/group) *p<0.05 *vs*. Control, **p<0.01 *vs*. Control, ***p<0.001 *vs*. Control, ^§^p<0.05 *vs*. Diabetic, ^§§^p<0.01 *vs*. Diabetic, ^§§§^p<0.001 *vs*. Diabetic.

In murine models, STZ-induced DM is associated with induction of pro-fibrotic genes and interstitial cardiac fibrosis. Therefore, to evaluate the role of DAPA in the prevention of cardiac fibrosis, profibrotic markers *Tgfb1*, *Pdgfb*, *Ccn2* (CTGF) and *Serpine1* (PAI-1) mRNA expressions were determined. Diabetic rats had an increased LV mRNA expression of aforementioned profibrotic markers. DAPA treatment diminished the elevation of *Tgfb1*, *Ctgf* and *Serpine1* (Fig 4A–4D). In parallel, myofibroblast marker α-SMA protein levels were upregulated in the diabetic group, which was lowered by DAPA (Fig 4E). Extracellular matrix (ECM) component fibronectin has a pivotal role in myocardial remodeling responses to pathological injury. DM-induced higher *Fn* expression was suspended by DAPA indicating milder myocardial remodeling and fibrogenesis (Fig 4F).

**Fig 5:**
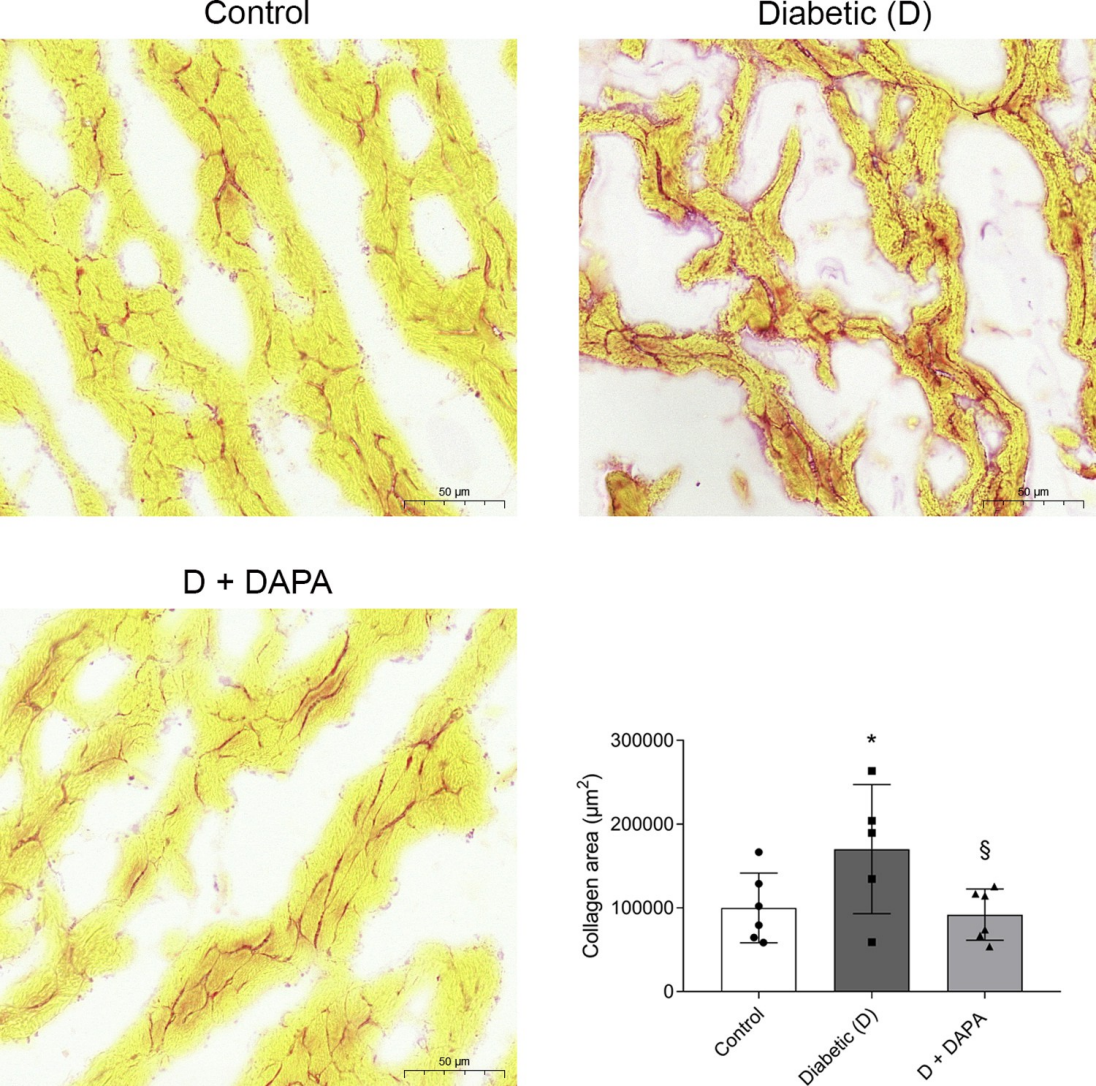
Diabetes-induced cardiac fibrosis is mitigated by DAPA treatment. Representative picrosirius red stained heart sections and quantitative evaluation of fibrosis of Control, diabetic (D) and dapagliflozin treated diabetic rats (D+DAPA). The red-stained area was measured based on the red color intensity. The myocardial field of measurement was manually selected with the caution of avoiding the endocardium and perivascular connective tissue. Original magnification, x400. Scale bar, 50 μm. Bars indicate means±SDs and data were analyzed by one-way ANOVA with Holm-Sidak multiple comparisons test (n = 5-6/group). *p<0.05 *vs*. Control, §p<0.05 *vs*. Diabetic.

Myocardial collagen content and perivascular fibrosis of the left ventricle was studied with picrosirius red staining. Diabetes-induced intramyocardial collagen deposition was detected. Collagen accumulation was reduced almost to control levels in the DAPA-treated group confirming its protective role and efficiency in decreasing cardiac fibrosis (Fig 5).

## Discussion

T1DM is associated with premature CVD and an increased risk of cardiovascular mortality [24]. Remodeling in the left ventricle is frequently observed in patients with T1DM and refers to structural changes associated with increased volume, myocyte hypertrophy, myofibroblast proliferation, and interstitial fibrosis. Thus, reversing or even preventing LV remodeling is an important target in the prevention of DCM. Our study provides experimental evidence that oral administration of SGLT2i DAPA over a 6-week period prevents metabolic decline, cardiac hypertrophy, and myocardial injury in STZ-induced T1DM. Reduction in aortic IMT suggests that DAPA could also prevent atherosclerosis. We also revealed that DAPA decreases the gene expressions related to LV hypertrophy, myocardial dysfunction, inflammation, and fibrosis indicating that DAPA alters the cardiac remodeling in diabetic rats.

The proof of cardiovascular safety for new glucose-lowering therapies has been required by US FDA since 2008. SGLT2i are a new class of antidiabetic medications showing reduction in the relative risk of HF in T2DM, as recently proven in EMPA-REG OUTCOME, CANVAS and DECLARE-TIMI 58, respectively [5–7]. The beneficial effects in cardiovascular outcome and mortality were also present in non-diabetic HF patients undergoing DAPA treatment [8]. SGLT2i reduce blood pressure, decrease body weight or induce natriuresis suggesting that consistent cardioprotection does not only relate to their antihyperglycemic action. Despite the encouraging results of these trials, the underlying molecular mechanisms of cardioprotection remain incompletely understood. Furthermore, the literary data is scarce about the effect of SGLT2i in T1DM.

Premature atherosclerosis is the main cause of mortality in T1DM with cardiovascular events occurring more than a decade earlier. Atherosclerosis begins early in life, but usually becomes symptomatic in adulthood. The build-up of fats, cholesterol, calcium in the arterial wall lead to arterial wall thickening and the development of arterial plaques [25]. Therefore, the prevention of atherosclerosis can be a crucial factor to reduce the risk of CVD later in life. Since earliest changes in vascular structure occur first in the abdominal aorta and IMT is an early marker of atherosclerosis, we measured aortic IMT of rats [26, 27]. Aorta of diabetic rats showed intimal thickening, irregularity and diffused elastic membranes, which were prevented by DAPA in T1DM. Only two non-randomized and one randomized studies evaluated the effects of SGLT2i on carotid IMT in T2DM patients. Empagliflozin reduced IMT, while ipragliflozin and tofogliflozin had no statistically significant alterations in IMT [28–30]. However, these studies have limitations: having small sample size and short treatment duration or not being a double-blind, placebo-controlled trial. A recent study reported that low serum klotho level is associated with larger thickness of carotid artery intima-media, thus it may be considered as an early predictor of atherosclerosis [31]. Here we found that DAPA reversed the decrement of serum klotho in T1DM. In parallel, in our study DM-induced serum total cholesterol-, triglyceride-, and LDL-C elevation were normalized to control levels in DAPA-treated rats, similarly to what was recently reported in T2DM murine models [32, 33]. According to this, SGLT2i may prevent atherosclerosis, however further investigations are needed to clarify the effects of SGLT2i on IMT.

Natriuretic peptides are predominantly produced in the atria and ventricles in response to increased cardiac stretch, thus they are powerful predictors of ventricular dysfunction and cardiovascular outcomes. Elevated ANP expression in adult ventricles is a marker for the development of hypertrophy, while BNP is predominantly released in response to LV volume expansion and pressure overload [34]. Levels of ANP and BNP are increased in diabetic patients, prediabetic and T2DM rat models [35–38]. Here, both natriuretic peptides were robustly elevated in the left ventricle of T1DM rats verifying LV dysfunction. Both *Nppa* and *Nppb* were less increased in DAPA-treated group, similar to what was recently observed in patients with T2DM [39, 40]. cTn are components of the contractile apparatus of myocardial cells and are accepted as standard biomarker for the diagnosis and prediction of myocardial injury [41]. cTnI is also elevated in HF patients with DM [42]. Canagliflozin possesses favorable effects on high‐sensitivity cTnI reduction in patients with T2DM [43]. Similarly, elevation of serum cTnI was observed in our T1DM experiment model, which was decreased by DAPA. Treatment with DAPA delayed the elevation of hypertrophy and myocyte damage markers indicating that SGLT2i may play a protective role in T1DM-induced ventricular remodeling.

Klotho proteins have received a great attention in the past few years as possible sensitive and specific markers for chronic kidney disease and CVD. Klotho proteins form a unique endocrine system that regulates multiple metabolic processes such as inactivation of oxidative stress, inflammation, and fibrotic pathways in the heart, cardiomyocytes, and kidney [44–46]. Recent studies reported that klotho level is lower in patients with T2DM and CVD supporting its protective role in DM-related cardiorenal diseases [47, 48]. The effect of SGLT2i on klotho in the serum has not been reported to-date, moreover only one study examined their connection in the kidney. Our finding demonstrates that DAPA halts the decrement of serum klotho in T1DM. Empagliflozin increased renal klotho expression in unilateral ureteric obstruction rat model [49]. Based on these findings, SGLT2i improve klotho levels independent of DM suggesting a novel mechanism of action in cardiorenal protection.

The link between the pathophysiology of DM and CVD is complex and multifactorial [50]. Inflammation has been implicated in the pathophysiology of both diseases. According to the ‘cytokine hypothesis’ HF is partly a consequence of activated endogenous cytokine cascades in the heart and circulation [51]. Pro-inflammatory cytokines (e.g. TNF-α, IL-1β) cause cardiac myocyte hypertrophy, contractile dysfunction, LV dilatation and modulate the interstitial matrix of the heart [52, 53]. Increased serum TNF-α and IL-6 levels of diabetic patients are associated with LV diastolic dysfunction [54]. In our experiment, elevation of *Il1b*, *Il6*, *Tnf* and *Ccl2* in the left ventricle indicate the activation of inflammatory signaling pathways, similarly to other T1DM models [55, 56]. We showed that DM-induced cardiac inflammation is diminished by DAPA treatment. This is in line with the anti-inflammatory effect of empagliflozin and DAPA observed in T2DM rodent models [16, 57]. Moreover, SGLT2i attenuated inflammation in infarcted rat hearts of APOE knockout mice and LPS mice model suggesting that cardioprotection does not solely depend on glucose lowering actions [15, 58, 59]. Together with literary data, our findings support the anti-inflammatory role of SGLT2i as a possible mechanism of action in the prevention of CVD.

Cardiac fibrosis is the common final pathway through which HF develops. Clinical studies showed the presence of myocardial fibrosis in patients with DM, which may occurs independently of hypertension or coronary atherosclerosis [60, 61]. Hyperglycemia-induced cardiac fibrosis provokes pathological changes that culminate in activation of TGF-β pathway and deposition of ECM proteins leading to increased myocardial stiffness and impaired cardiac function [62]. Recent studies showed that SGLT2i attenuate cardiac fibrosis in T2DM rodent models and TGF-β-induced fibroblast activation in human cardiac fibroblasts [63–65]. We previously reported that DAPA has antifibrotic effects in T1DM kidneys, therefore it seemed plausible that DAPA prevents fibrosis in the heart as well [66]. Here we found that elevated *Tgfb1*, and *Ccn2* expressions were reduced in DAPA-treated rats. Further, we showed that accumulation of α-SMA, *Fn* and collagen were also mitigated after DAPA treatment supporting the antifibrotic potential of SGLT2i. These results suggest that SGLT2 inhibition, independent of the type of DM, may have propitious effects on cardiac TGF-β pathway, thus myocardial fibrosis.

In conclusion, the current study provides experimental data for the cardioprotective effect of DAPA in experimental T1DM. Our results indicate that DAPA prevents intimal thickening, cardiac inflammation, and fibrosis. All these mechanisms suggest that a complex system lies behind the organoprotective effect of SGLT2i. Ultimately, various SGLT2i may provide a novel therapeutic opportunity to treat and improve outcomes of DM and related disorders simultaneously.

## Study limitation

Cardiovascular function (e.g., hemodynamics, echocardiography) measurements were not performed during the animal experiment due to the lack of equipment in our laboratory.

## Supporting information

S1 Table Dapagliflozin treatment did not affect control animals. Metabolic parameters of control, dapagliflozin-treated control (C+DAPA) rats at the end of the 6-week experimental period. Values are presented as means±SDs and data were analyzed by one-way ANOVA with Holm-Sidak multiple comparisons test (n = 6/group). **p<0.01 *vs*. Control. UN: undetectable, LDL-C: low-density lipoprotein cholesterol, GOT: serum glutamate-oxaloacetate transaminase, GPT: serum glutamate-pyruvate transaminase.
(PDF)

S2 Table Mean arterial pressure, heart rate and heart to body weight ratio. Mean arterial pressure, heart rate and heart to body weight ratio of control, dapagliflozin-treated control (C+DAPA) rats. Values are presented as means±SDs and data were analyzed by one-way ANOVA with Holm-Sidak multiple comparisons test or Kruskal-Wallis with Dunn comparison test (n = 6/group).
(PDF)

S1 Data (PDF)

## Abbreviations

α-SMA: Alpha-Smooth Muscle Actin
ANP: Atrial Natriuretic Peptide
BNP: B-Type Natriuretic Peptide
CTGF: Connective Tissue Growth Factor
cTn: Cardiac Troponins
cTnI: Cardiac Troponin I
cTnT: Cardiac Troponin T
CVD: Cardiovascular Disease
DAPA: Dapagliflozin
DCM: Diabetic Cardiomyopathy
DM: Diabetes Mellitus
ECM: Extracellular Matrix
FDA: Food and Drug Administration
FGF: Fibroblast growth factor
FN: Fibronectin
HF: Heart Failure
IL-1β: Interleukin-1β
IL-6: Interleukin-6
IMT: Intima-Media Thickness
LV: Left Ventricular
MAP: Mean Arterial Pressure
MCP-1: Monocyte Chemoattractant Protein 1
Nlrp3/ASC: (NOD) like receptor protein 3/ apoptosis-associated speck
PAI-1: Plasminogen Activator Inhibitor 1
PDGF: Platelet-Derived Growth Factor
Rn18S: 18S Ribosomal RNA
SGLT2i: Sodium-Glucose Cotransporter 2 Inhibitors
STZ: Streptozotocin
T1DM: Type 1 Diabetes Mellitus
T2DM: Type 2 Diabetes Mellitus
TGF-β: Transforming Growth Factor Beta
TNF-α: Tumor Necrosis Factor Alpha
