# Supplementary material for: SGLT2 inhibitor dapagliflozin prevents atherosclerotic and cardiac complications in experimental type 1 diabetes
Source: PLoS One. 2022 Feb 17;17(2):e0263285. doi: 10.1371/journal.pone.0263285 (PMC8853531; doi:10.1371/journal.pone.0263285)
Supplement: S1 Table — Metabolic parameters of control, dapagliflozin-treated control (C+DAPA) rats at the end of the 6-week experimental period. Values are presented as means±SDs and data were analyzed by one-way ANOVA with Holm-Sidak multiple comparisons test (n = 6/group). **p<0.01 vs. Control. UN: undetectable, LDL-C: low-density lipoprotein cholesterol, GOT: serum glutamate-oxaloacetate transaminase, GPT: serum glutamate-pyruvate transaminase. (PDF) [file pone.0263285.s001.pdf]

S1 Table. Dapagliflozin treatment did not affect control animals.

Metabolic parameters	Control (C)	C+DAPA
Body weight (g)	442±35.4	414±34.9
Non-fasting glucose (mmol/L)	6.42±0.58	5.60±0.62
Fructosamine (μmol/L)	143±3.74	143±8.45
Total cholesterol (mmol/L)	1.96±0.15	1.82±0.21
Triglycerides (mmol/L)	1.24±0.51	1.05±0.48
LDL-C (mmol/L)	0.44±0.15	0.47±0.09
GOT (U/L)	127±19.6	195±30.9
GPT (U/L)	43.0±8.39	49.2±11.3
Glucosuria	UN	114±0.60**

Metabolic parameters of control, dapagliflozin-treated control (C+DAPA) rats at the end of the 6-week experimental period. Values are presented as means±SDs and data were analyzed by one-way ANOVA with Holm-Sidak multiple comparisons test (n = 6/group). **p<0.01 *vs.* Control. UN: undetectable, LDL-C: low-density lipoprotein cholesterol, GOT: serum glutamate-oxaloacetate transaminase, GPT: serum glutamate-pyruvate transaminase.
