# Supplementary material for: SGLT2 inhibitor dapagliflozin prevents atherosclerotic and cardiac complications in experimental type 1 diabetes
Source: PLoS One. 2022 Feb 17;17(2):e0263285. doi: 10.1371/journal.pone.0263285 (PMC8853531; doi:10.1371/journal.pone.0263285)
Supplement: S2 Table — Mean arterial pressure, heart rate and heart to body weight ratio of control, dapagliflozin-treated control (C+DAPA) rats. Values are presented as means±SDs and data were analyzed by one-way ANOVA with Holm-Sidak multiple comparisons test or Kruskal-Wallis with Dunn comparison test (n = 6/group). (PDF) [file pone.0263285.s002.pdf]

S2 Table. Mean arterial pressure, heart rate and heart to body weight ratio.

	Control	C+DAPA
Mean arterial pressure (mmHg)	88.5±3.66	86.2±3.44
Heart rate (bpm)	444±12.9	399±24.2
Heart to body weight ratio (%)	0.29±0.01	0.31±0.05

Mean arterial pressure, heart rate and heart to body weight ratio of control, dapagliflozin-treated control (C+DAPA) rats. Values are presented as means±SDs and data were analyzed by one-way ANOVA with Holm-Sidak multiple comparisons test or Kruskal-Wallis with Dunn comparison test (n = 6/group).
