# Supplementary material for: SGLT2 inhibitor dapagliflozin prevents atherosclerotic and cardiac complications in experimental type 1 diabetes
Source: PLoS One. 2022 Feb 17;17(2):e0263285. doi: 10.1371/journal.pone.0263285 (PMC8853531; doi:10.1371/journal.pone.0263285)

Western blot gel images

α-SMA Western blot gel images

Control * Control * IC Diabetic D+DAPA

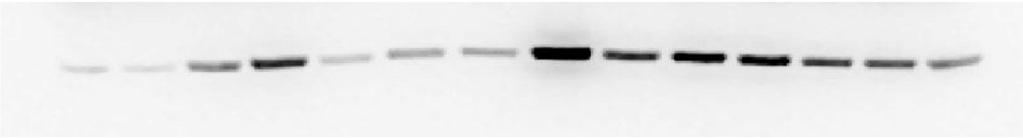

Membrane 1

← 42 kDa

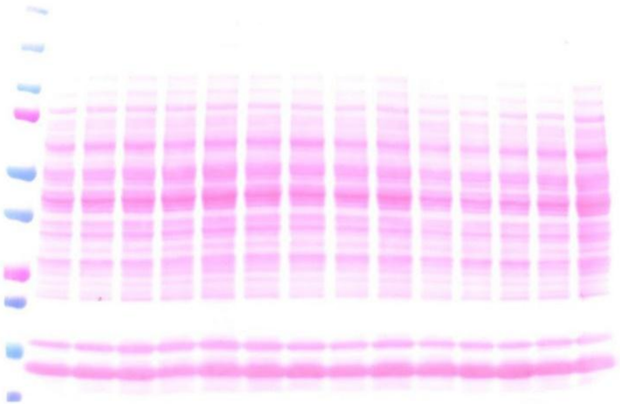

Diabetic * * IC D+DAPA # D+DAPA * D+DAPA

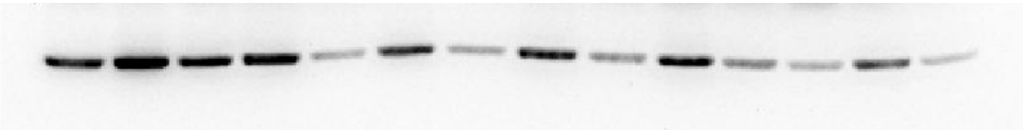

Membrane 2

← 42 kDa

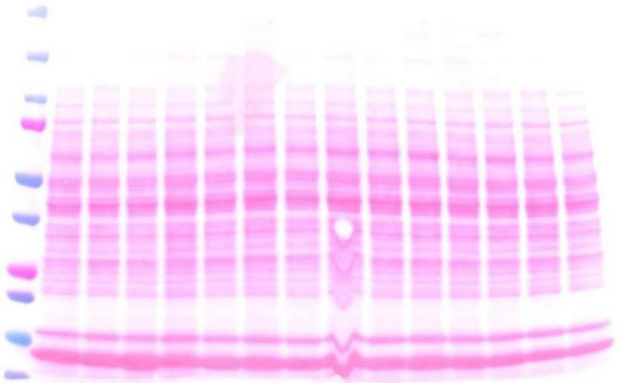

Inner control is an equal mixture of all samples measured in this experiment, loaded onto each gel in order to be able to compare them.

In order to be able to measure several different proteins from the same samples, the membranes were cut into slices. Therefore, wider original gel images can not be provided .

IC: Inner control
*: Samples from another experiment. Data not used for this study.
#: Technical problem

Quantitative RT-PCR melting curves

Il1, Il6, Tnf and *Ccl2* melting curves

Il1

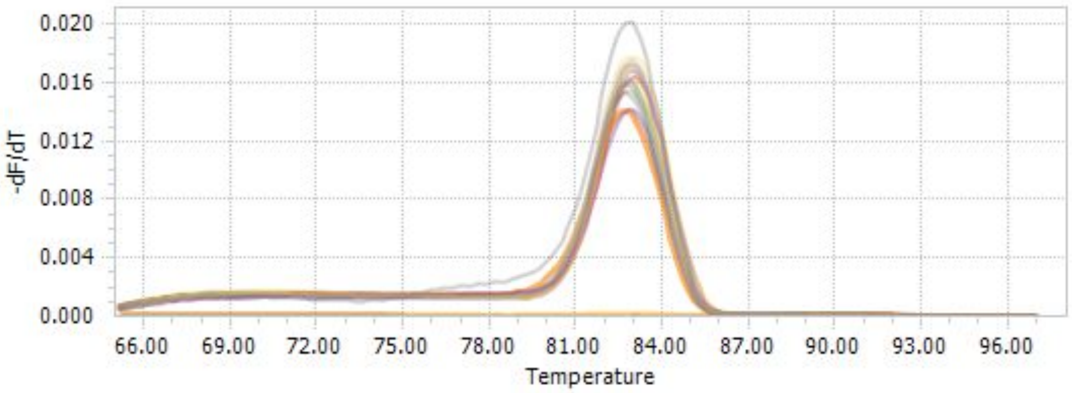

Il6

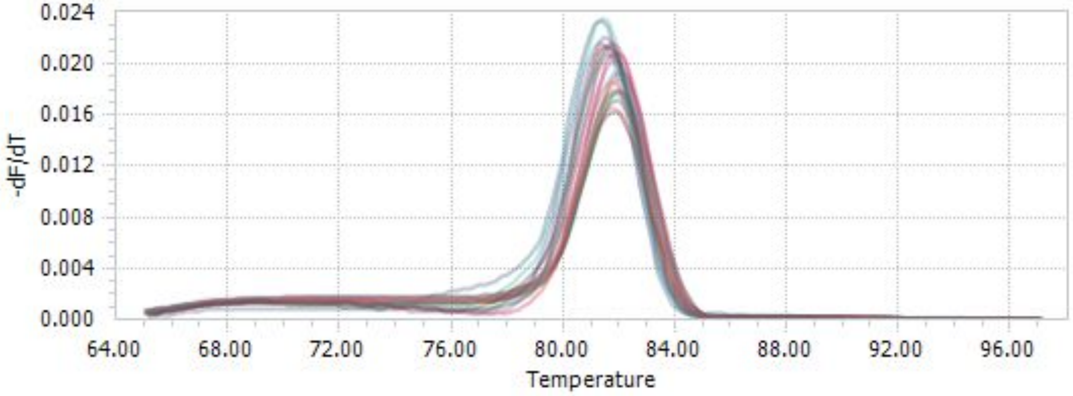

Tnf

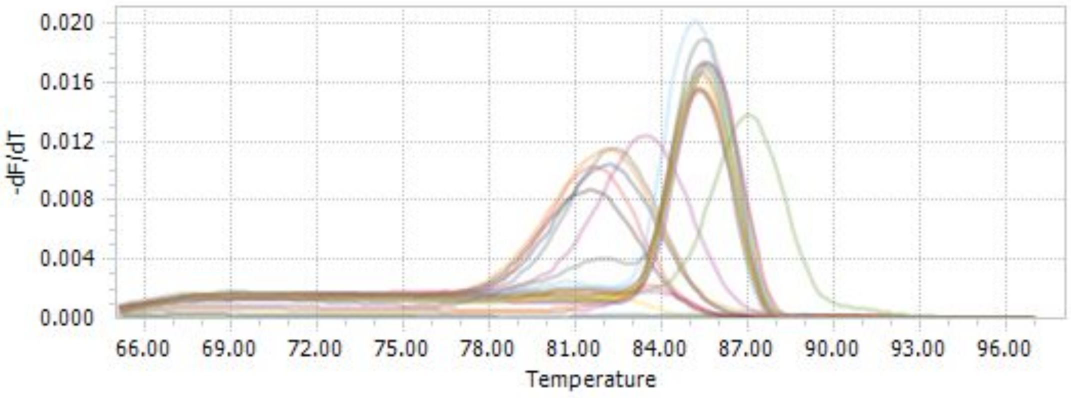

Ccl2

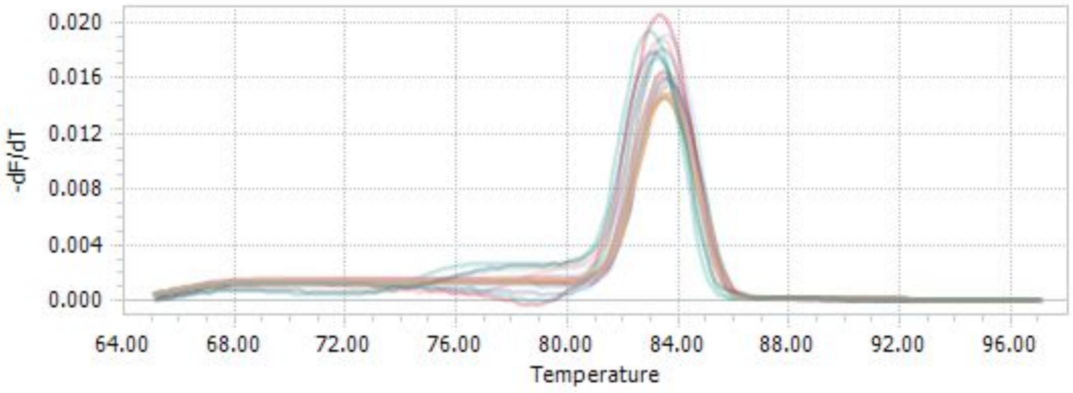

Tgfb, *Pdgf* and *Ccn2* melting curves

Tgfb

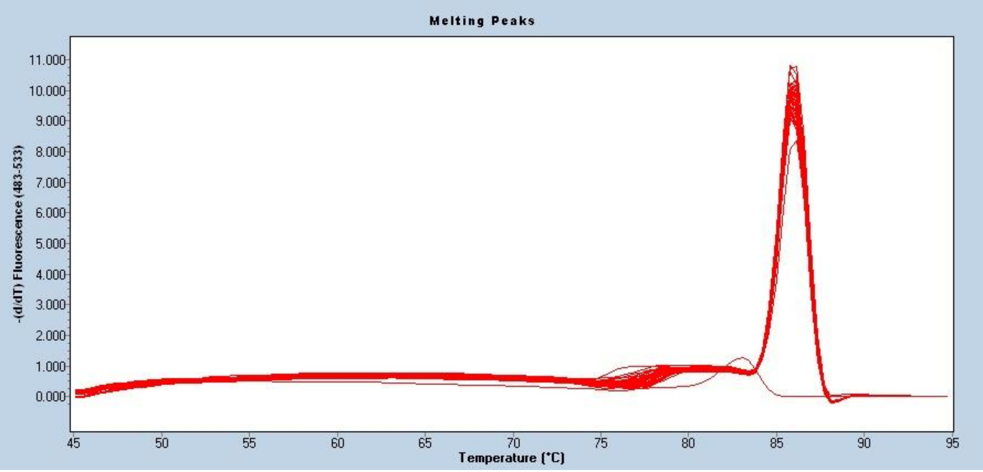

Pdgf

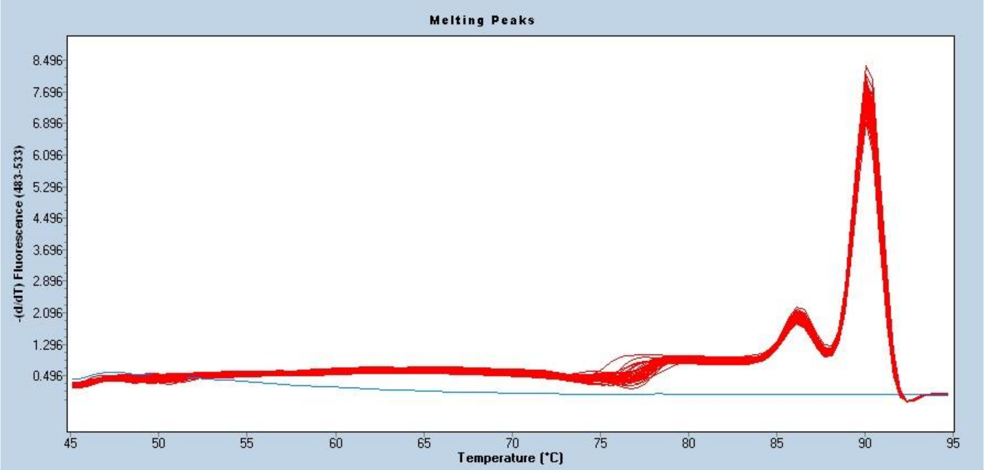

Ccn2

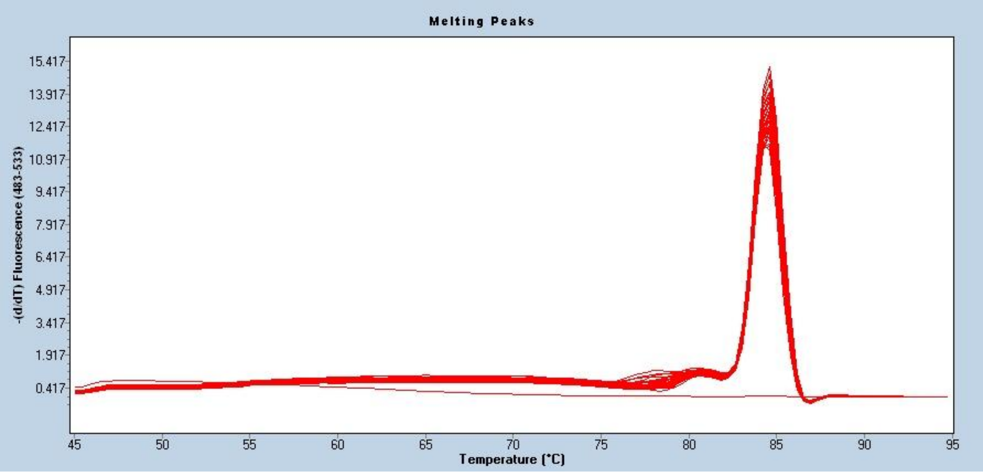

Serpine1 and *Fn* melting curves

Serpine1

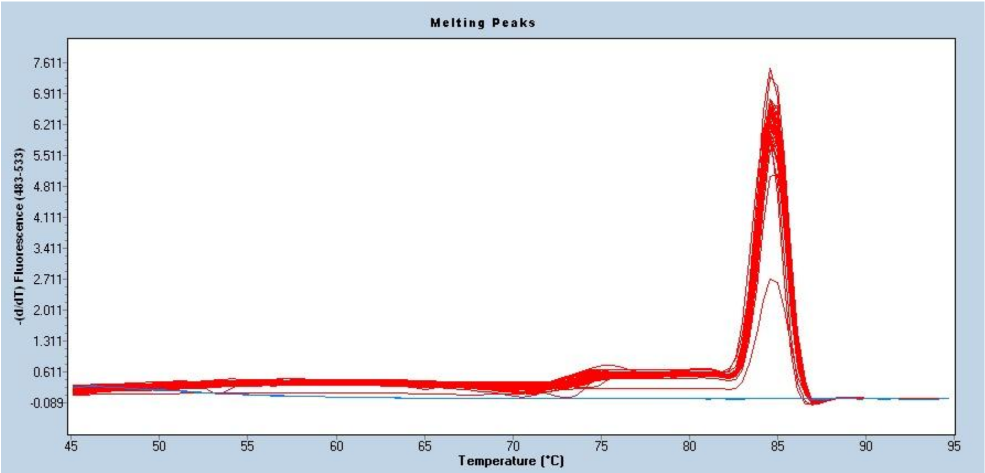

Fn

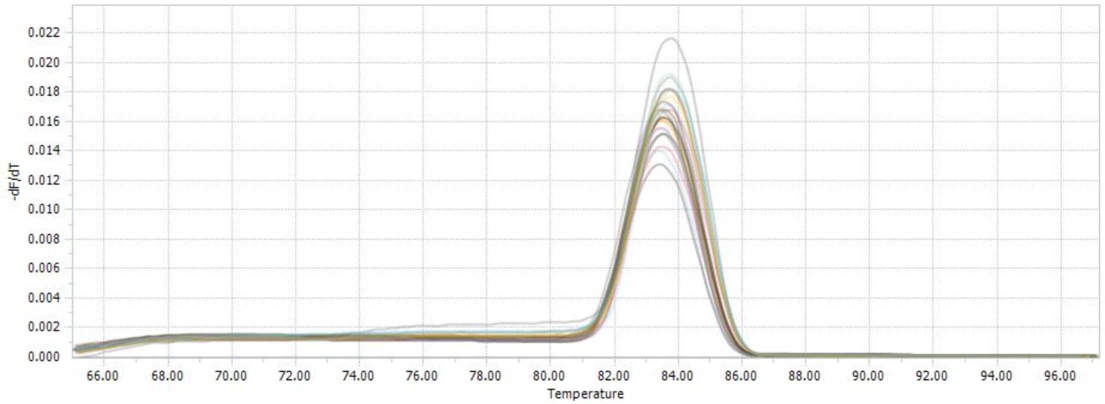

Nppa and *Nppb* melting curves

Nppa

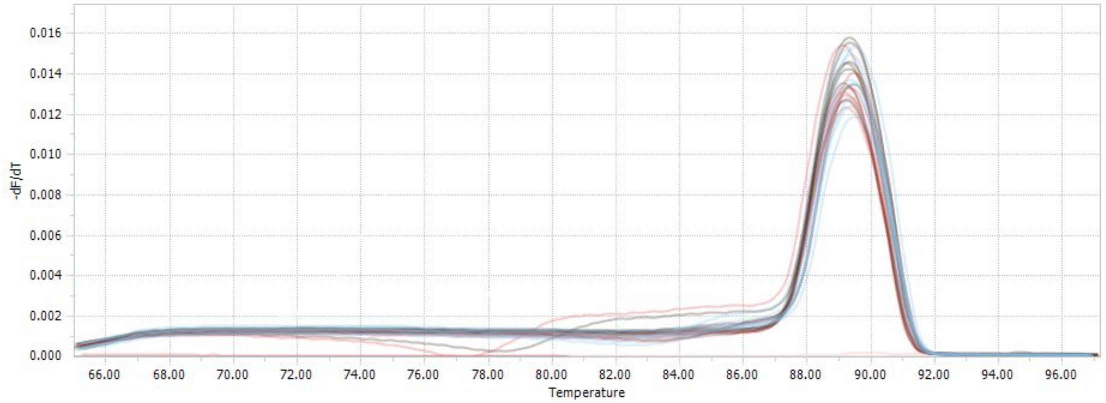

Nppb

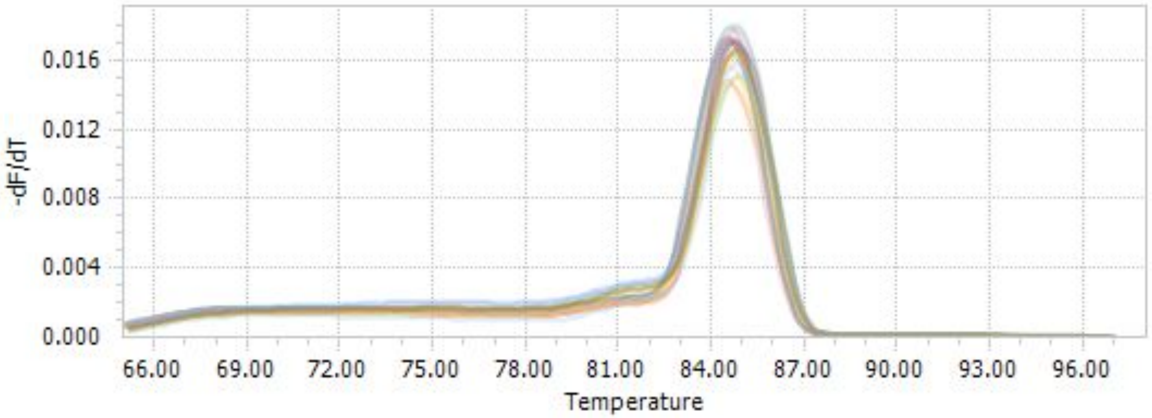

Supplement: S1 Data — (PDF) [file pone.0263285.s003.pdf]
